# Hyper-Methylated Hub Genes of T-Cell Receptor Signaling Predict a Poor Clinical Outcome in Lung Adenocarcinoma

**DOI:** 10.1155/2022/5426887

**Published:** 2022-04-06

**Authors:** Zixin Hu, Chongxiang Xue, Jiabin Zheng, Xingyu Lu, Jia Li, Huijing Dong, Yixuan Yu, Xu Zhang, Kexin Tan, Huijuan Cui

**Affiliations:** ^1^Department of Oncology, China-Japan Friendship Hospital, Yinghuayuan East Street 2, Chaoyang, Beijing, China; ^2^Beijing University of Chinese Medicine, No. 11, 3rd Ring Road East, Chaoyang, Beijing, China

## Abstract

**Background:**

Immune checkpoint inhibitors (ICIs) emerge as the first-line treatment of lung adenocarcinoma (LUAD); selection of subpopulations acquiring clinical benefit is required. Associations between epigenetic modulation of tumor microenvironment (TME) and clinical outcome are far from clear. We focused on immune-related genes closely regulated by DNA methylation to identify the potential clinical outcome indicators.

**Methods:**

We systematically calculated immunophenotype score (IMpS) and classified immunophenotypes based on seven TME features in three independent cohorts. The overlapping of differential expressed genes and methylated probes targeted genes was regarded as genes closely regulated by DNA methylation. Then, probe/gene pairs which highly correlated with each other and IMpS were identified and named as immune-related probe/gene pairs (mIMg). Prognostic mIMg were selected and verified in seven independent validation cohorts.

**Results:**

Three immune phenotypes were clustered, and similar results were obtained in the three independent training cohorts. C2 displayed as an immunologically hot phenotype, whereas C3 corresponded with immunologically cold phenotype. Average methylation level was decreased from C2 to C3 (C2 > C1 > C3). Similarly, ICIs nonresponders showed global hypo-methylation compared with responders. Genes in mIMg were mainly enriched, especially in T-cell receptor activation, and repressed in noninflamed TME by hyper-methylation. Among mIMg, low expression and hyper-methylation of CD247, LCK, and PSTPIP1 were risk factors of overall survival (OS). ICIs nonresponders were more likely to be hyper-methylated in the three genes. By integrating with the oncogenes status, we demonstrated that EGFR wt and SRGN overexpressed patients were associated with chronic inflammation and immune evasion, showing an immunologically hot phenotype, which might lead to the short OS but derive clinical benefit from ICIs.

**Conclusions:**

This study identifies hyper-methylation and concurrent repression of CD247, LCK, PSTPIP1 as immune negative indicators and risk factors for prognosis in LUAD. Moreover, EGFR/SRGN axis may participate in immune modification to influence ICIs response and clinical outcome in LUAD.

## 1. Introduction

Nonsmall cell lung cancer (NSCLC) is the main subtype of lung cancer, which accounts for the primary cause of cancer-associated mortality. The treatment of lung adenocarcinoma (LUAD), the most common form of NSCLC, has achieved great progression in the last ten years with more and more targetable oncogene alternations identified [1]. What is more, immune checkpoint inhibitors (ICIs) targeting the programmed cell death protein 1 (PD-1) and its ligand (PD-L1) axis have markedly changed the first‐line treatment of LUAD without molecular targets. Currently, extensive challenges still exist as only a subset of patients deriving clinical benefit [2].

Numerous studies had proved that the heterogeneity of tumor immune feature discriminates the clinical outcome of immunotherapies. The disturbance of immune effectors and regulators in the tumor microenvironment (TME) is just like the soil, fostering cancer cells (seeds) and participating in tumor development and progression. Dynamic equilibrium exists as the determinant of tumor immunogenicity, including the balance between infiltration of effectors (activated CD8+/CD4+ T-cells and T effector memory (Tem) cells) and suppressors (regulatory T (Treg) cells and myeloid-derived suppressor cells (MDSCs)), the dominating type of T helper cells (Th1 and Th2), and the expression of co-stimulators and co-inhibitors [3]. The immunophenotypes of TME predict response to ICIs. Immune-inflamed (also known as immunologically hot) TME was usually characterized by highly infiltrating T-cells accompanied by expression of suppressive molecules, such as PD-L1. The inflamed tumors profoundly respond well to ICIs. However, immune-excluded and immune-deserted (noninflamed or immunologically cold) TMEs are always associated with T-cell exclusion and unsusceptible to immunotherapy [4, 5].

DNA methylation is a well-known epigenetic process, which participates in cancerogenesis and has been reported as a promising biomarker in the diagnosis of NSCLC [6, 7]. With the widespread of ICIs, the relationship between TME and DNA methylation gradually grabbed much attention. TME shapes the fate of tumors with the aid of epigenetic alternations to modulate the dynamic gene expression. Moreover, DNA methylation silences or activates hub genes in the pivot immune signaling to shape TME and vice versa [8, 9]. Although challenges exist, managing immune response to predict ICIs treatment outcome is a routine trend and outlook. So far, combinatorial analyses of epi-immune signatures have preliminarily declared the ability to predict survival and ICIs response [10]. Fewer hyper-methylated hub genes in critical ICIs response pathways correlated with improved ICIs response [11, 12]. A simple prognostic profile or measure panel is an increasingly popular option in the routine use. However, even for the commonly and originally used biomarkers of ICIs response, such as PD-L1 status and tumor mutation burden (TMB), there are several exceptions for the differentiation between responders and non-responders. The predictive single hub genes in ICIs response merit further research. Hence, clinically available biomarkers for optimizing the use of ICIs and understanding the molecular determinants of immune response are still needed [13].

In this study, by integrating epigenetic and transcriptomic information of three LUAD cohorts, we explored the DNA methylation pattern of different immune phenotypes and identified hub genes of immune response which were regulated by DNA methylation and potentially influenced clinical outcome.

## 2. Methods

### 2.1. Data Collection and Processing

Three gene expression profiles of LUAD were downloaded as training cohorts. TCGA-LUAD level 3 RNA-seq data (HTSeq-Counts) was directly downloaded by using the GDC data transfer tool (https://portal.gdc.cancer.gov/). GSE60644 (Illumina HumanHT-12 V4.0 expression bead chip), GSE66863 (Agilent-028004 SurePrint G3 Human GE 8 F0B4 60 K Microarray) were downloaded from Gene Expression Omnibus (GEO) datasets (https://www.ncbi.nlm.nih.gov/). HTSeq-Counts were transformed into log 2 transformed transcripts per kilo-base per million mapped reads (TPM). Gene length was calculated as the sum of lengths of nonredundant exon. Illumina nonnormalized summary-level data were read by “read.ilmn” function and normalized by the “neqc” function. Agilent single channel microarray intensity data were read by “read.maimages” function and processed by “backgroundCorrect” and “normalizeBetweenArrays” unction. The abovedescribed processes were carried out by limma package [14].

The corresponding DNA methylation data (IDATs) including TCGA-LUAD, GSE56044, and GSE66836 methylation beta value were obtained [15, 16]. Collectively, the three datasets were measured with Illumina HumanMethylation450 bead chip. Raw data were read by “read.metharray.exp” function to found rgSet [17] and filtered by “champ.filter” function. Specially, poor performing probes with detection *p* value more than 0.05, belonging to sex chromosome, known to have common SNPs at the CpG sites, or having been demonstrated to map to multiple places in the genome were removed prior to differential methylation analysis [18]. Normalization was performed by BMIQ method with “champ.norm” function. The DNA methylation pipeline was realized by minfi [17] and ChAMP [18] R package.

Five cohorts including GSE37745, GSE50081, GSE14814, GSE41271, and GSE42127 were utilized for the validation of prognosis associated genes [19–23]. Two cohorts with oncogene status including GSE13213, GSE11969 were used for verification analysis grouped by oncogene status [24, 25]. DNA methylation cohorts containing ICIs response including GSE119144 and its corresponding expression data GSE135222 were used for the exploration of association between ICIs response and methylation/expression status. The raw files of Affymetrix were normalized by affy R package with Robust Multi-array Average (RMA) algorithm [26]. The procedures of processing Illumina and Agilent data were consistent with the training cohorts. Expression data from the same microarray manufactory were combined and the “ComBat” function in sva package was applied to adjust for batches deriving from different gene sets [27].

Minus germ-line somatic copy number alternations (sCNA) and merged somatic simple nucleotide variations (sSNV) segmented data of TCGA-LUAD cohort were downloaded from GDAC Firehose (Broad Institute TCGA Genome Data Analysis Center, https://gdac.broadinstitute.org/) for oncogene status analysis. Data utilized for our study were listed in Supplementary Table S1 and have been uploaded to Github (https://github.com/HU-ZX/01_mIMg_LUAD_2022).

### 2.2. Estimation of TME and Classification of Immunophenotype Clusters

For each LUAD sample in the training cohorts, we first quantified the immune activity of tumor by single sample gene set enrichment analysis (ssGSEA) [28, 29]. Immune features were measured by the following categories: (1) inflitration of immune effectors (aCD4 + T, aCD8 + T, CD4 + Tem, CD8 + Tem) (2) immune suppressors (Tregs and MDSCs) (3) dominating T helper cells type (Th1 and Th2) (4) expression of MHC molecules (5) expression of co-stimulators and co-inhibitor [3]. Then, we weighted ssGSEA scores of positive immune factors (MHC molecules, effectors, Th1 cells, co-stimulators) by “+1” and negative immune factors (suppressors, Th2, co-inhibitors) by “−1” to calculate immune activity scores (IMaS) of every feature. Unsupervised hierarchical cluster was performed based on the IMaS of seven features and immunophenotype score (IMpS) was calculated by the sum of IMaS of every feature. The gene list containing 28 immune cell populations, MHC molecules, and immunostimulatory and immunoinhibitory factors was retrieved from the publication [3, 30]. The consistency of different clusters was verified by cytolytic activity (CYT), which represented the ultimate effective mechanism in the cancer immunity cycle and was calculated as the geometric mean of granzyme A (GZMA) and perforin (PRF1) expression levels as previously defined [31]. The fraction of infiltrating cells were calculated using methylation data by EpiDISH [32]. The fraction of CD3+ T-cells (CD4+ T-cells and CD8+ T-cells) and epithelial cells were compared among different clusters.

### 2.3. Identification of Immune-Related Genes Closely Regulated by DNA Methylation

Microarray probes annotation files were directly downloaded from GEO datasets. Homo. sapiens GRCh38.p13 GFF3 (v35) file downloaded from GENCODE website (https://www.gencodegenes.org/) was used for id transforming. Gene symbol was used as gene identification. If gene ID did not map one-to-one to the gene symbol, the first annotated gene was used to represent the others. RNA-seq data were preprocessed by normalizing distributions with “calcNormFactors” function in edgeR [33] and transforming to log2-counts per million (CPM) by the “voom” function in limma [14]. Linear modelling and empirical Bayes moderation in limma [14] were carried out to identify different expression genes (DEGs) of three datasets. Genes with *p*value less than 0.05 and the absolute value of log-2-fold-change (FC) more than 0 were considered as DEGs.

The average methylation level of each sample was calculated as mean of beta value of all methylation probes. Different methylation probes (DMPs) of three datasets were calculated using linear models implemented in ChAMP [18]. Probes with adjust *p*value less than 0.05 and the absolute value of log-2-fold-change (FC) more than 0 were considered as DMPs.

For assessing genes that underwent transcriptional regulation by gaining DNA methylation, we selected the overlapping of DEGs and DMPs corresponding genes, which were more likely to be different genes regulated by DNA methylation and named as mDEGs. Spearman correlation between methylation beta value and gene expression was carried out. Besides, the correlation between gene expression and DNA methylation beta value with IMpS was also performed. Probe/gene pairs whose correlation coefficient was no less than 0.5 and *p* value less than 0.05 were selected. The procedures described above were repeated in the three training cohorts, and the overlapping probe/genes were regarded as ultimate results. Probe/gene pairs which met the following requirements were regarded as the immune-related probe/gene pairs (mIMg): (1) the pairs were closely related with each other (|*r*| > 0.5, p.val <0.05); (2) both the probes and genes in the pairs were highly correlative with IMpS (|*r*| > 0.5, p.val <0.05. Beta value of each probe in mIMg was used for principal components analysis (PCA) in the three training cohorts by “PCA” function in FactoMineR package.

### 2.4. Functional Enrichment Analysis

We further analyzed functions of the mDEGs. Gene Oncology (GO) functional enrichment was carried out in biological processes (BP), cellular components (CC), and molecular functions (MF). Terms with a *p*value < 0.05 were regarded as significant. The enrichment score was calculated as previously reported to combine the number and status (upregulated or downregulated) of genes enriched in the pathway [34]. Candidate genes were used for further stratification of overall population. Gene Set Enrichment Analysis (GSEA) was used to excavate mechanisms of them. Ontology gene sets in MSigDB were used for analysis [35]. Protein–protein interaction network (PPI) was performed by STRING website (https://string-db.org). Association between proteins encoded by genes in mIMg was represented by combined scores, which suggested the result of combinatorial analysis of gene neighborhood, fusion, occurrence, co-expression, and some other parameters [36]. R packages including clusterProfiler [37], GSVA, and ggplot2 were used for analysis and visualization.

### 2.5. Statistical Analysis

Statistical analysis was conducted with R software (version 4, 4.0.4). The bioinformatic processes could be repeated with the R scripts uploaded on https://github.com/HU-ZX/01_mIMg_LUAD_2022. The datasets used in the study were showed in Table S1 and original data are accessible to download from the link attached in Github page. ANOVA test or Kruskal Wallis H test was used for continuous variables in multiple groups. T test or Mann Whitney U test was used for comparison between two groups. Differences between distributions of the groups were estimated by the Chi-squared test. Expression data and methylation beta value were directly used for the assessment of mIMg status. Overall, survival time (OS) and vital status of LUAD patients were used to represent clinical outcome. Patients were divided into two groups based on the threshold of each candidate marker. The thresholds were selected by “surv-cutpoint” function implemented in survminer R package to decrease the batch effect of calculation. Probes and genes in mIMg were, respectively, brought into multivariate Cox analysis by adjusting gender, age, stage to select potential prognostic biomarkers. The Kaplan–Meier method was used to estimate OS, and log-rank test between groups were performed. Hazard ratios (HRs) were derived from univariate Cox regressions of potential prognostic probe/gene biomarkers. Statistical significance was set as *p* < 0.05. C-index based on univariate Cox model was calculated using the R package survcomp [38]. The receiver operating characteristic (ROC) curves at years 3, 5, and 9 years of the multivariate Cox model including age, gender, stage, and candidate molecular factors were used to evaluate the discriminative ability of molecular indicators using the R package timeROC [39]. Efficiency of candidate molecular biomarkers was assessed by decision curve analyses [40].

## 3. Results

### 3.1. Unsupervised Immunophenotype Clusters

**Figure 1:**
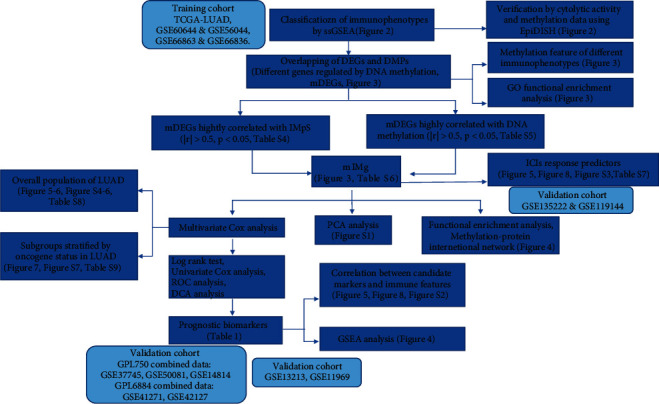
Workflow of this study.

**Figure 2:**
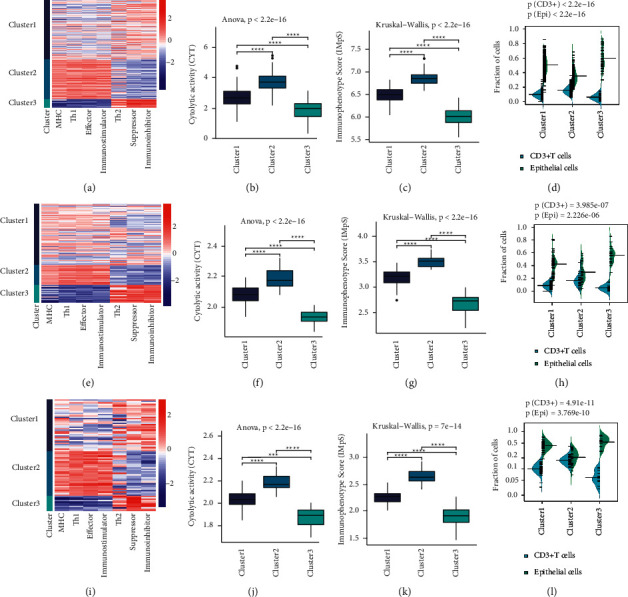
Classification of immunophenotypes. (a, e, i) Heatmaps of immune activity scores (IMaS) based on seven immune features in the TCGA-LUAD, GSE66863 & GSE66836, GSE60644 & GSE56044 cohort, IMaS was calculated based on ssGSEA scores. Positive immune factors (MHC molecules, effectors, Th1 cells, co-stimulators) were weighted by “+1,” whereas negative immune factors (suppressors, Th2, co-inhibitors) were weighted by “−1.” Both positive and negative immune factors were all over-expressed in C2 cluster and shaped C2 as immunologically hot phenotype. C3 was, on the contrary, showed as immunologically cold phenotype. Immune features of C1 were heterogeneous. Samples in C1 were not as distinctive as those in C2 and C3, so we named the group of patients as immunologically medium. (b, f, j) Comparison of Cytolytic activity (CYT) among different immunophenotype clusters, C2 cluster elicited the strongest immune clearance response. (c, g, k). Comparison of IMpS of immunophenotype clusters in the 3 training cohorts. Immunophenotype score (IMpS) was calculated as the sum of IMaS of seven features. (d, h, l) Beanplots of infiltrating CD3+ (CD4+ and CD8+) T-cells and epithelial cells, which were calculated by EpiDISH based on methylation data. C2 cluster had the highest CD3+ T-cells, and C3 had the most epithelial cells.

The workflow of this study is shown in Figure 1. Basic information and clinical pathological features of training and validation cohorts used in this study are showed in supplementary tables Table S2 and Table S3, respectively. There were 450 patients in the TCGA cohort, 112 patients in the GSE66863 & GSE66836 cohort, 78 patients in the GSE60644 & GSE56044 cohort for the integrative analysis of transcriptomic and epigenetic data. Samples were clustered into three clusters based on the IMaS of 7 immune features respectively in the above-described cohorts. Similar cluster results were obtained in different datasets and showed by the heatmaps (Figures 2(a), 2(e), and 2(i)).

Both immune effectors and regulators were in the dominant position in the C2 cluster. In detail, tumors of the C2 cluster were characterized by the presence of CD8+ and CD4+ T-cells which act as defenders in the proximity of tumors. However, the immunosurveillance system in C2 was also activated which manifested as high expression of co-inhibitors including immune checkpoints and other immunosuppressed factors. C2 cluster presented as preexisting anti-tumor immune response, tending to be immunologically hot phenotype, and was more likely to benefit from immunotherapies.

In contrast, the C3 cluster was characterized by fewer infiltrated TILs and low expression of immune regulators, which was more likely to be immunologically cold or noninflamed tumor. Not surprisingly, tumors in the C3 cluster belonged to ICIs-resistant subset. C1 cluster demonstrated high immune heterogeneity and could not be classified into C2 or C3 cluster (Figures 2(a), 2(e), 2(i)).

IMpS, which represented the immune response of tumor, decreased from C2 to C3 (C2 > C1 > C3, Kruskal–Wallis test, *p* < 2.2*e* − 16, *p* < 2.2*e* − 16, *p*=7*e* − 14, Figures 2(c), 2(g), 2(k)). Besides, CYT showed the same trend (C2 > C1 > C3, Kruskal–Wallis test, *p* < 2.2*e* − 16, *p* < 2.2*e* − 16, *p*=7*e* − 14, Figures 2(b), 2(f), 2(j)). Fractions of infiltrating cells including CD3+ (CD4+ and CD8+) T-cells, epithelial cells were calculated based on the methylation data. CD3+T-cells decreased from C2 to C3 (C2 > C1 > C3, *p* < 2.2*e* − 16, *p* < 3.985*e* − 07, *p*=4.91*e* − 11, Figures 2(d), 2(h), 2(l)). Epithelial cells were in the opposite trend (C2 > C1 > C3, *p* < 2.2*e* − 16, *p*=2.226*e* − 06, *p*=3.769*e* − 10, Figures 2(d), 2(h), 2(l)). Comparison between two groups in the three cohorts was all significant. Tumors in C2 and C3 cluster had discriminative immune features; therefore, DNA methylation pattern of them were compared in the following analysis.

### 3.2. Methylation Features of Different Immunophenotypes

**Figure 3:**
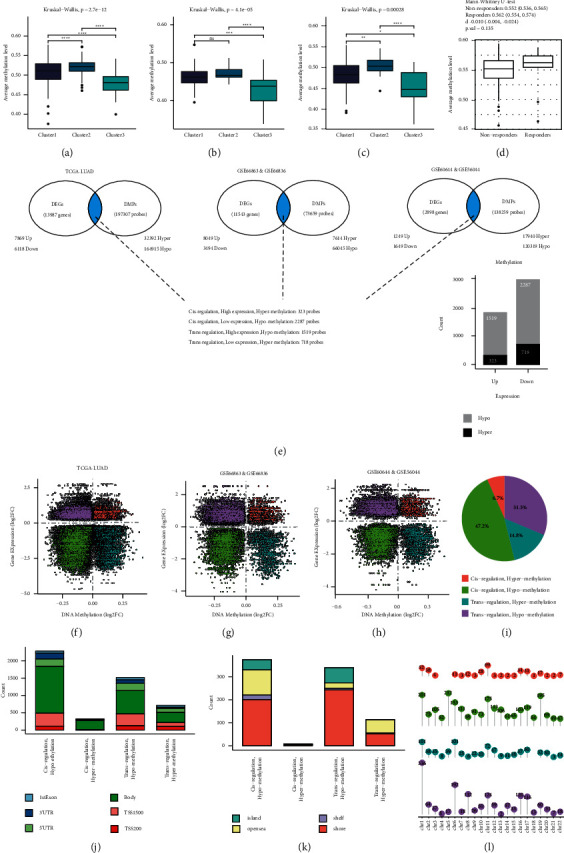
DNA methylation features of immunophenotype clusters. (a–c) Comparison of average methylation level among different clusters in the training cohorts. (d) Comparison of average methylation level between ICIs responders and nonresponders. (e) Overlapping of DEGs and DMPs corresponding genes in the three training cohorts (mDEGs). (f–h) Patterns of genes regulated by DNA methylation in the three training cohorts. (i) Proportion of DNA methylation regulation patterns. (j, k) Distribution of DNA methylation probes in different gene regions. (l) Chromosome location of DNA methylation probes.

The average beta value of each sample was used for assessing the global methylation features. Notably, the average methylation level was consistent with the trend of IMpS (C2 > C1 > C3, Kruskal–Wallis test, *p*=2.7*e* − 12, *p*=0.00028, *p*=4.1*e* − 05, Figures 3(a)–3(c)), which validated the result published before [9]. The same trend was observed in the ICIs responders and nonresponders, albeit no significant difference was observed (Figure 3(d)).

C3 (noninflamed tumor cluster) and C2 (inflamed tumor cluster) had different immune features; 4847 overlapping probe/gene pairs of DEGs and DMPs among the three cohorts were identified (Figures 3(e)–3(h)). 3806 probes were hypo-methylated in a noninflamed tumor. The cis-regulatory pattern was defined as hypo-methylation repressed gene expression or hyper-methylation activated gene expression, in which condition, the changing trend of gene expression and DNA methylation level was the same. The trans-regulatory pattern was defined as hyper-methylation repressed gene expression or hypo-methylation activated gene expression. In our study, we confirmed that DNA methylation was more likely to trans-regulate gene expression (Chisq test, X-squared = 25, *p* = 2.035*e* − 07, Figure 3(i)). DNA methylation was unevenly distributed. TSS1500 contained more hypo-methylated probes in C3 compared with non-TSS1500 region (Chisq test, X-squared = 28.08, *p* = 1.164*e* − 07, Figure 3(j)). Probes located in promoter (TSS200, TSS1500, 1^st^exon, 5'UTR) were more likely to trans-regulate gene expression while those located in gene body tended to cis-regulate gene expression (Chisq test, X-squared = 202.34, *p* < 2.2*e* − 16, Figure 3(j)). Flanking regions of CG island (CGI), also known as “shore,” were enriched with more trans-regulative probes compared with CGI region (Chisq test, X-squared = 51.129, *p* = 8.648*e* − 13, Figure 3(k)). The chromosomal distribution characteristics of probes were presented in Figure 3(l).

### 3.3. Hyper-Methylation of Hub Genes in Immune Response Predicted Noninflamed Phenotype

**Figure 4:**
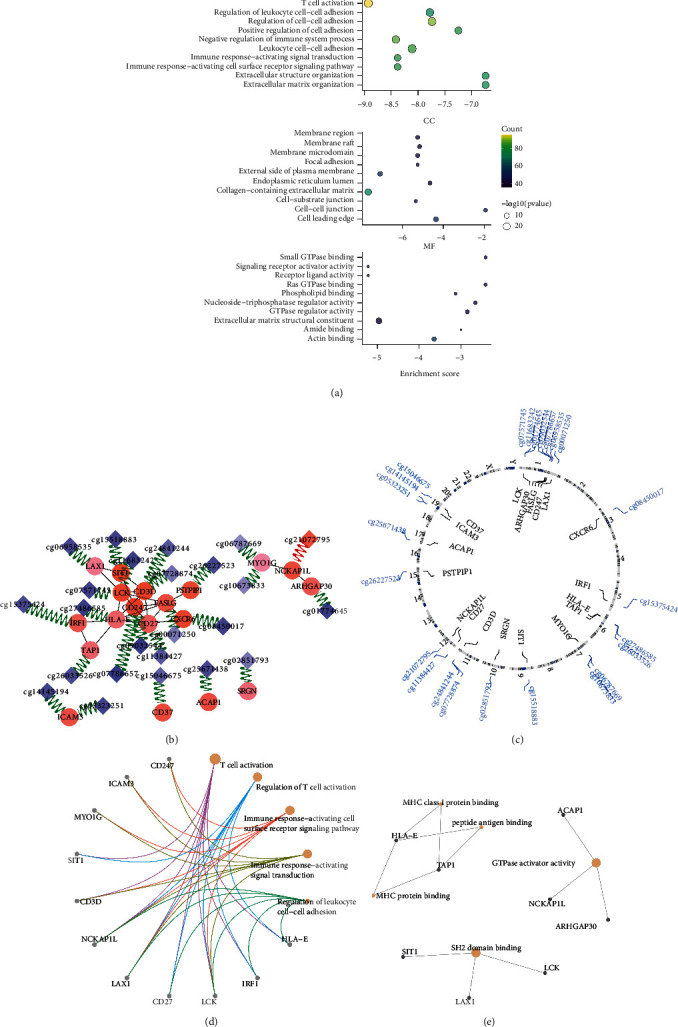
Excavation of mIMg. (a) Gene oncology (GO) analysis of mDEGs. Terms including cell component (CC), molecular functions (MF), and biological processes (BP) were analyzed. (b) Correlation among genes and probes in mIMg. Circles represent proteins encoded by genes in mIMg, while diamonds represent probes in mIMg. Continuous color of circles and diamonds indicates the Spearman correlation coefficient of the gene (probe) and IMpS. Purple means negative correlation and red represents positive correlation. Gray lines connecting two circles represent correlation among proteins, the thickness of lines represent the combined scores calculated by STRING, which suggest the strength of the interactions. Green or Red lines connecting a circle and a diamond denote the Spearman correlation coefficient between gene and probes. Green represents trans-regulative pattern and red indicates cis regulation. (c) Circular plot indicates the chromosome distribution of probe/gene in mIMg. (d, e) Association between genes in mIMg and corresponding enrichment in BP and MF processes.

Functional enrichment analysis showed that the mDEGs enriched in T-cell activation and some other immune-related processes. Besides, it suggested GTPase and Src homology 2 (SH2) domain as potential targets of DNA methylation regulatory system in immunophenotype. GTPase is a rate-limiting enzyme in GTP hydrolysis to participate in G protein inactivation. SH2 domain is a special structure in the combination of enzymes and prolongs docking time. The two are in the downstream of TCR activation signaling. The processes described above were all downregulated in the C3 cluster (Figure 4(a)).

Spearman correlation identified 168 probes and 86 corresponding genes which highly correlated with IMpS (Supplementary Table S4). Meanwhile, correlation between methylation level and gene expression was performed, and 61 probes whose beta level were highly correlated with its corresponding gene expression were selected (Supplementary Table S5). Ultimately, 24 probes and 19 corresponding genes which were contained in both two probe/gene sets described above were defined as mIMg (Supplementary Table S6, Figures 4(b) and 4(c)). PCA analysis of three training cohorts indicated that mIMg successfully distinguished patients with different immunophenotypes in the three LUAD training cohorts (Figures S1(a)–S1(c). Trans-regulative pattern was more common in mIMg and genes in mIMg tended to be repressed by hyper-methylation in the noninflamed tumor (Supplementary Table S6, Figures 4(b) and 4(c)). Functional enrichment analysis of the 19 genes demonstrated that mIMg operated an important position in T-cell activation. The crucial structures in transduction of T-cell activation were identified, including GTPase and SH2 domain (Figures 4(d) and 4(e)).

Methylation level of IMpS-related mDEGs according to ICIs response in the GSE119144 cohort were investigated, and 44 of 168 probes were significantly different between responders and nonresponders with the same trend in the training cohort (Supplementary Table S7-1). 10 probes were in mIMg, including cg07786657, cg09032544, cg07728874, cg24841244, cg11384427, cg08450017/, cg11683242, cg14145194, cg15518883, and cg26227523. However, expression of the corresponding genes was not significantly different (Supplementary Table S7-2).

### 3.4. Silence of T-Cell Activation Associated Genes by DNA Methylation Predicted a Poor Clinical Outcome

To investigate the prognostic capacity of mIMg, multivariate Cox analyses adjusted for age, gender, stage based on hyper/hypo methylation (high/low expression) groups of the probe/gene pairs were performed, respectively, in the TCGA-LUAD cohort, and 15 probes together with their corresponding 13 genes distinguished OS (Supplementary Table S8). Prognostic ability of genes in mIMg was verified in two integrative expression cohorts (Supplementary Table S3). ArfGAP with coiled coil, ankyrin repeat and PH domains 1 (ACAP1), Rho GTPase-activating protein 30 (ARHGAP30), CD247, lymphocyte transmembrane adaptor 1 (LAX1), lymphocyte-specific protein tyrosine kinase (LCK), proline-serine-threonine phosphatase interacting protein 1 (PSTPIP1) stood the test (Table 1). Among these prognostic mIMg factors, hyper-methylation and concurrent downregulation of gene expression was significantly blamed for low OS.

**Figure 5:**
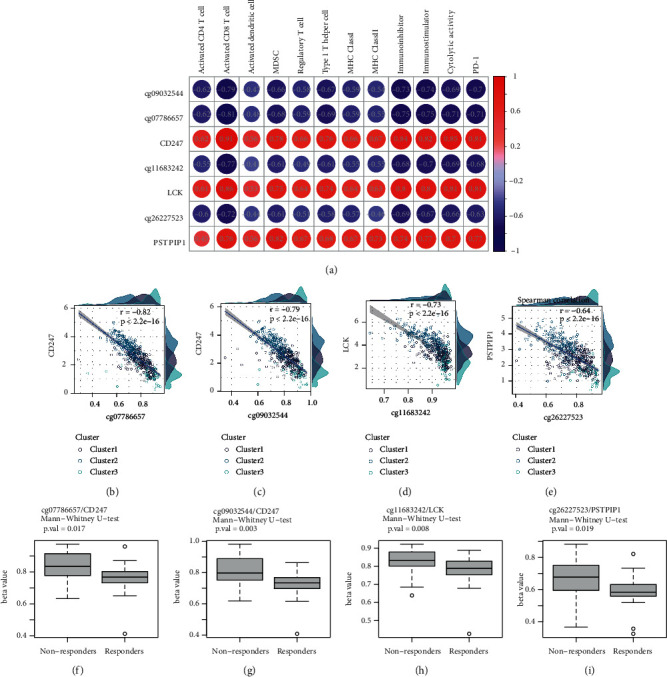
Hyper-methylation and low expression of immune-related hub genes predicting weak immune response. (a) Correlation between cg09032544/CD247, cg07786657/CD247, cg11683242/LCK, cg26227523/PSTPIP1 in mIMg and seven immune features in TCGA cohort. CD247, LCK, and PSTPIP1 were all positively correlated with immune response, whereas methylation probes of the three genes were negative factors. (b–e) Scatter plots of correlation between methylation probe and gene. Cg09032544, cg07786657, cg11683242, and cg26227523 trans-regulated their corresponding genes. (f–i) Comparison of cg09032544, cg07786657, cg11683242, and cg26227523 methylation level between ICIs responders and nonresponders in GSE119144.

**Figure 6:**
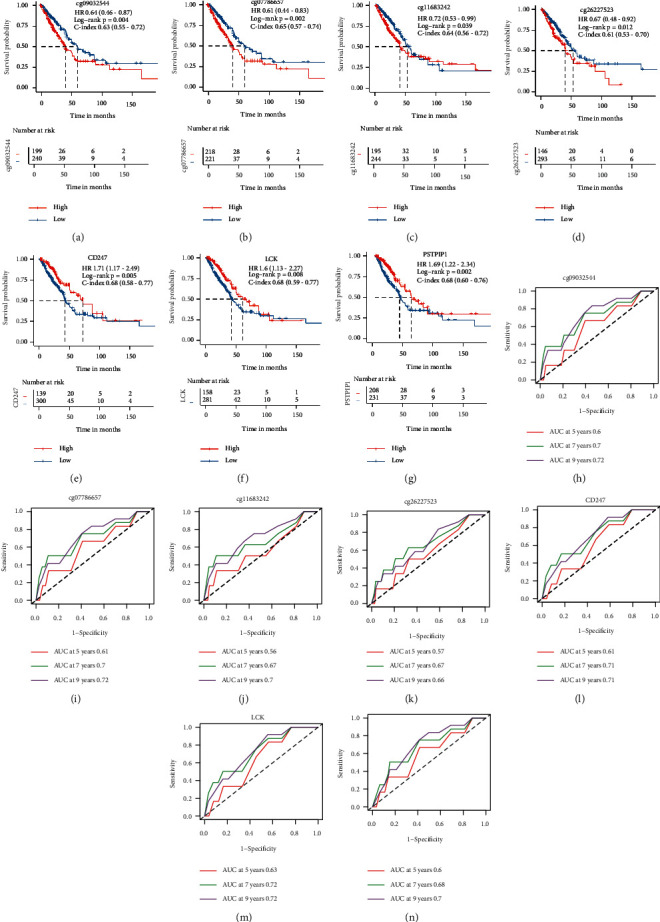
Hyper-methylation and low expression of immune-related hub genes predicting poor prognosis. (a–g) Comparison of overall survival (OS) between groups according to methylation/expression level of cg09032544/CD247, cg07786657/CD247, cg11683242/LCK, and cg26227523/PSTPIP1 in TCGA-LUAD cohort. C-index and hazard ratio (HR) were calculated by univariate Cox analysis of methylation/expression level group. (h–n) ROC curves based on multivariate Cox models combining molecular factors (cg09032544/CD247, cg07786657/CD247, cg11683242/LCK, and cg26227523/PSTPIP1), age, gender, and stage in the TCGA-LUAD cohort.

Notably, cg09032544/CD247, cg07786657/CD247, cg11683242/LCK, and cg26227523/PSTPIP1 separated not only immune response but also survival time. CD247, LCK, PSTPIP1 are hub genes in T-cell activation, whose methylation level were closely correlated with expression level (Figures 5(b)–5(e)). Hypo-methylation of cg09032544, cg07786657, cg11683242, and cg26227523 were protective factors of ICIs response and OS (Figures 5(f)–5(i), Figures 6(a)–6(d)). Concurrently, low expression of CD247, LCK, PSTPIP1 were risk factors in OS (Figures 5(e)–5(g)), and the results were validated in GPL570 cohort and GPL6884 cohort (Supplementary Figures S4(A)–S4(C), S4(G)–S4(I)). However, expression of the three genes had no significant difference between responders and nonresponders in GSE135222 (Supplementary Figures S3(A)–S3(C)). C-index based on univariate Cox model and ROC curves based on multivariate Cox model indicated that the seven prognostic factors could be used as discriminative tool for predicting prognosis of LUAD (Figures 6(h)–6(n), Supplementary Figures S4(D)–S4(F), S4(J)–S4(L)). Decision curves analysis of multivariate Cox models containing molecular prognostic stratified factor (CD247 or LCK or PSTPIP1), age, gender, stage was performed to further explore the predictive efficiency of the three genes. The analysis was successfully achieved in TCGA-LUAD training cohort and GPL6884 integrative validation cohort (Supplementary Figures S5(A) and S5(B), S5(D) and S5(E)) but failed in GPL570 integrative cohort for batch effects. To conclude, silence of CD247, LCK, and PSTPIP1 by hyper-methylation might repress T-cell activation and affect ICIs response and survival. Expression level of the three genes could be used as prognosis indicators in LUAD.

ACAP1 and ARHGAP30 were closely correlated with immune infiltration parameters. The two were GTPase-activating proteins (GAPs) which might act in the downstream T-cell activating pathway (Supplementary Figure S6(A)), albeit no difference was observed in their expression and methylation level according to ICIs response (Supplementary Figures S6(B)–S6(E)). Hypo-methylation of ACAP1 and ARGAPs were protective factors for OS, whereas over-expression of the two genes were risk factors (Supplementary Figures S6(F)–S6(M)).

### 3.5. Crosstalk between DNA Methylation and Oncogenic Mutations

Growing evidence suggests that Epidermal Growth Factor Receptor (EGFR) and other oncogenic driver mutations modify the TME with low immune checkpoints and TMB thus reduce ICIs response [41]. More mechanisms associated with ICIs resistance are gradually surfacing and DNA methylation is one. Average methylation level of three clusters stratified by EGFR status, KRAS status, and tumor protein p53 (TP53) status was consistent with total population, Supplementary Figures S7(A)–S7(F)).

**Figure 7:**
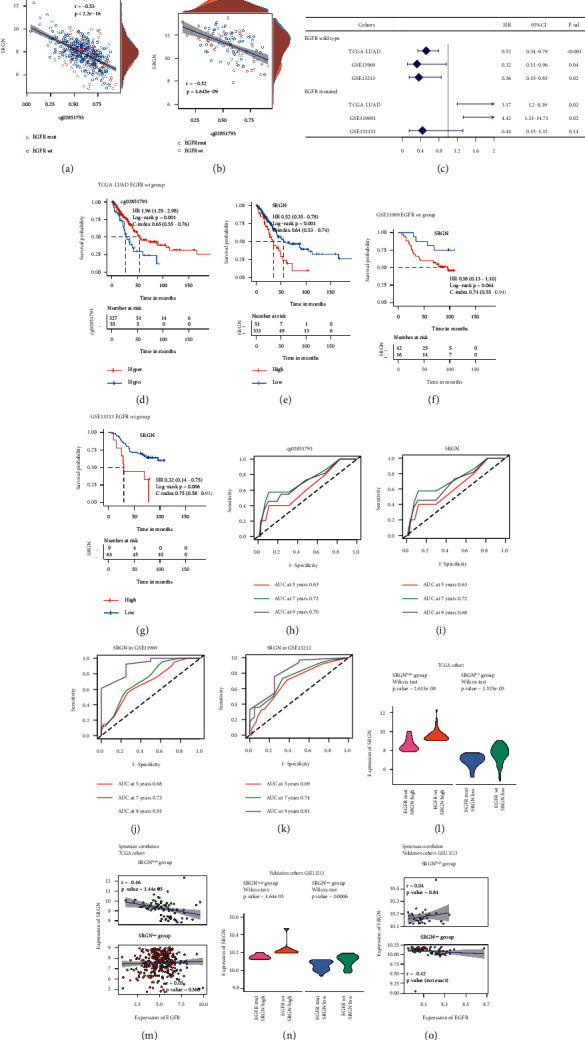
EGFR/SRGN axis potentially involved in TME modification. (a, b) Scatter plots between beta values of cg0285179 and expression of SRGN. SRGN were negatively regulated by methylation regardless of EGFR status in TCGA cohort and GSE66863 and GSE66836 cohorts. (c) Forest plot of SRGN stratified by EGFR status in the training and validation cohorts. (d, e) Comparison of overall survival (OS) between groups based on methylation/expression level of cg02851793/SRGN in TCGA-LUAD EGFR wt cohort. C-index and hazard ratio (HR) was calculated by univariate Cox analysis of methylation/expression level group. (f, g) Validation of SRGN discriminating OS in EGFR wt group of GSE11969 and GSE13213 cohorts. (h, i) ROC curves based on multivariate Cox model combining molecular stratification factors (cg02851793/SRGN), age, gender, stage in TCGA-LUAD EGFR wt cohort. (j, k) ROC curves of SRGN in EGFR wt group of GSE11969 and GSE13213 cohorts. (l), (n) Comparison of SRGN between EGFR wt and EGFR mut groups in SRGN ^high^ and SRGN ^low^ groups. EGFR wt group tended to express more SRGN. (m, o) SRGN was negatively regulated by methylation despite EGFR status.

Multivariate Cox analyses were carried out according to different oncogene status, respectively, in TCGA-LUAD cohort. IMpS-related mDEGs whose expression and methylation level concurrently had prognostic ability were used for further multivariate Cox validations in GSE11969 and GSE13213 cohorts (Supplementary Table S9). Although prognostic mIMg was found in EGFR mut, KRAS mut/wt, TP53 mut/wt group, these genes did not stand validation tests. mDEGs which potentially influenced prognosis were presented in the forest plots (Supplementary Table S9, Figures S7(G)–S7(I)). cg02851793/Serglycin (SRGN) was a prognostic pair according to EGFR status. Expression of SRGN was regulated by methylation regardless of EGFR status (Figures 7(a) and 7(b)). The total group was stratified into SRGN ^high^ and SRGN ^low^ groups based on OS. Low expression of SRGN and hyper-methylation in cg02851793 was a protective factor in the EGFR wt group of TCGA-LUAD cohort (Figures 7(c)–7(e)). Low expression of SRGN also predicted a better OS in the EGFR wt group of GSE11969 and GSE13213 cohorts (Figures 7(c), 7(f) and 7(g)). However, low expression of SRGN was a risk factor in the EGFR mut group in TCGA-LUAD and GSE11969 but failed to be validated in GSE13213 (Figure 7(c)). Methylation level of SRGN did not affect survival in the EGFR mut group. Log-rank test was used to compare OS between SRGN over-expressed and low-expressed group. C-index based on univariate model of SRGN was calculated in EGFR wt patients (Figures 7(d)–7(g)). ROC curves based on multivariate cox model were used to evaluate the prognostic ability in training and validation cohorts (Figures 7(h)–7(k)).

**Figure 8:**
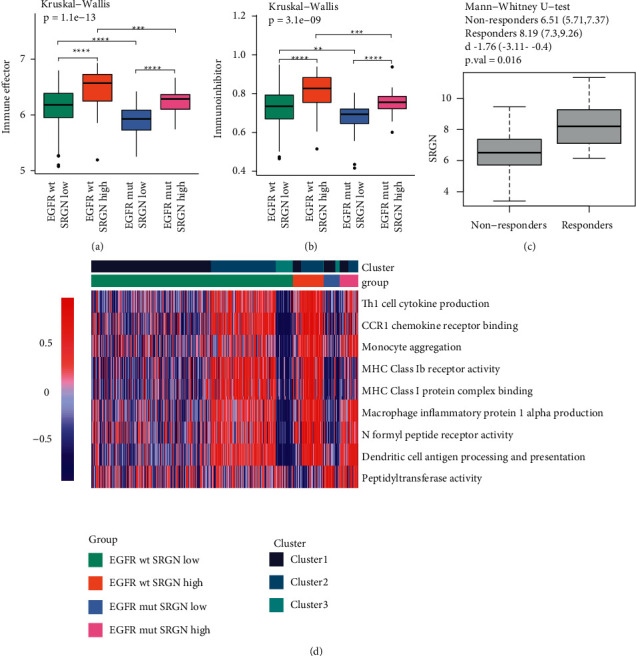
(a, b) Comparison of immune effectors (aCD4+T, aCD8+T, CD4+Tem, CD8+Tem) and immunoinhibitors regarding EGFR status and SRGN expression level. (c) Comparison of SRGN between ICIs responders and nonresponders. (d) Heatmap of GSEA analysis based on EGFR status and SRGN expression level.

We demonstrated that the expression level of SRGN cooperating with EGFR status could discriminate clinical outcome. Then, we explored the expression of SRGN and EGFR status. Although comparison of SRGN in EGFR mut and wt groups was not significant in TCGA cohort and GSE13213 validation cohort (*p*=0.7341, *p*=0.09173), SRGN was higher expressed in EGFR wt group in both SRGN ^high^ and SRGN ^low^ groups (Figures 7(l), 7(n)). The expression SRGN and EGFR tended to be low correlated with each other, yet it was not validated successfully (Figures 7(m), 7(o)). GSEA analysis was carried out to identify potential mechanisms. Notably, all patients in EGFR wt and SRGN high group shared inflamed phenotypes (C1 and C2). Immune effective cells were highly infiltrating accompanied by expression of immune inhibitors in this group (Figures 8(a) and 8(b)). MHC-I antigen presentation process and Th1 cells activity were enriched in this group (Figure 8(d)). Moreover, initiate immune pathways were also enriched, indicating chronic inflammation (Figure 8(d)). In contrast, EGFR mut and SRGN low group was characterized as a noninflamed phenotype. EGFR wt and SRGN low group showed immune heterogeneity and was well classified by immune cluster. In addition, SRGN was higher in ICIs responders compared with nonresponders, which confirmed that SRGN might function to modify TME potentially (Figure 8(c)). To sum up, with the involvement of immune disturbance, EGFR wt and SRGN high patients were associated with short OS but might benefit from ICIs.

## 4. Discussion

In recent years, ICIs have achieved impressive success in the treatment of LUAD. Selection of responders is becoming a more and more crucial issue. There is a growing awareness that cancer cells are fostered by highly heterogeneous and plastic cells in TME engaging in well-orchestrated reciprocal interactions [42]. Immunologically hot or inflamed TME is characterized with high density of immune effectors accompanied by the expression of immune checkpoints. In this case, ICIs reinvigorate anti-tumor immune response. In contrast, immunologically cold or noninflamed TME lacks ICIs response [4]. In our study, three immune phenotypes were classified according to seven immune features; C2 cluster corresponded to immunological hot phenotype, indicating preexisting anti-tumor immune response, and was more likely to benefit from immunotherapies. C3 cluster had fewer infiltrated TILs accompanied by low expression of immune regulators, tending to be an immunologically cold or noninflamed tumor. Tumors in C3 cluster were more likely to be ICIs-resistant subset.

DNA methylation is a key regulatory strategy in epigenetic modulation of gene expression. It was previously reported that hyper-methylation on the promoters of tumor suppressor genes could promote tumorigenesis [43, 44]. Emerging evidences have suggested the critical role of DNA methylation in immune modification of tumors and mediating the immune response [8, 9]^.^ Recent studies had demonstrated that DNA methylation loss counteracted with TMB and copy number load formed by genome instability in mitotic cell division and involved in immune evasion to increase ICIs resistance [45]. We confirmed that noninflamed TME and ICIs nonresponders underwent methylation loss. The result implies that the combinatorial regimen of DNA methyl-transferase inhibitors (DMTis) and ICIs holds promise for improving the therapeutic efficacy of ICIs [46].

At present, biomarkers are continuing to be excavated for directing ICIs response as target therapy. Emerging studies are developing prediction models for immune response or clinical outcome [4]. Herein, we focused on genes involving in immune modification which are closely regulated by DNA methylation, naming these methylation probe/gene pairs as mIMg. Although the average methylation level was low in the immunologically cold cluster and ICIs nonresponders, hub genes in immune-related pathways, especially in T-cell receptor activation, were silenced by hyper-methylation.

As we all know, two distinct steps must be completed to elicit an effective anti-tumor response. Firstly, dendritic cells (DCs) accomplish antigen cross-presentation for CD8+ T-cell initiation. Then, antigens must be directly presented by the tumor via MHC class I pathway and recognized by activated CD8+ T-cells for killing [5]. Among mIMg, most genes in noninflamed tumor were repressed by hyper-methylation and involved in CD8+ T-cell activation, especially in the process of interaction between MHC class I and T-cell receptor (TCR) complex. TAP1 encodes antigen peptide transporter 1, which involves in the transport of antigens from the cytoplasm to the endoplasmic reticulum for association with MHC class I molecules. CD247, CD3D encode zeta and delta chains of CD3 which participate in TCR complex. Zeta chains transduce positive signals by immunoreceptor tyrosine-based activation motifs (ITAMs). Apart from editing of TCR, the downstream signal also experienced hyper-methylation, such as LCK, which encodes Syk family kinases Lck and associates with CD4 or CD8 cytoplasmic tails. LCK phosphorylates the tyrosine residues of ITAMs in CD3 and zeta chains and transduces the TCR activation signal in an SH2 domain-dependent manner [47]. Moreover, the hyper-methylation of co-stimulators and regulators in the CD8+ T-cell activation such as intercellular adhesion molecule 3 (ICAM3), PSTPIP1, CD27, CD37, signaling threshold regulating transmembrane adaptor 1 (STI1), LAX1, Myosin IG (MYO1G) also proved to participate in immunologically cold phenotype. We then compared methylation/expression level of mIMg according to ICIs response for validation, and found that the methylation level of CD247, CD3D, LCK, ICAM3, STI1, PSTPIP1, and CXCR6 were enhanced in the nonresponders, which verified our discovery. Even so, no significant difference was found in the expression of genes described above. As 58 samples in GSE119144 had complete methylation data whereas only 27 samples in the corresponding expression cohort GSE135222 had transcriptomic data, we speculated that the failure of distinction between responders and nonresponders in transcriptomic level could come down to the small sample size. Moreover, as epigenetic modulation occurred earlier than expression change, methylation changes might be more sensitive than expression changes.

To investigate the clinical value in genes repressed by hyper-methylation in noninflamed TME, multivariate Cox analyses adjusted for universally known prognostic factors (gender, age, stage) were carried out among mIMg. We demonstrated that repression of CD247, LCK, PSTPIP1 by hyper-methylation corresponded with immunological cold phenotype and were risk factors of OS. The crucial role of CD247 and LCK need not to be restated more. PSTPIP1 encodes a CD2 cytoplasmic tail-binding protein and acts as an immunoinhibitor by blocking CD2/CD58 contact. CD2 interacts with CD58 at the initial stages of T-cell activation even prior to the recognition of TCR and MHC by increasing the dwell time between antigen presentation cells (APC) and T-cell [48]. In our study, we suggested that hyper-methylation and concurrent repression of CD247, LCK, and PSTPIP1 attenuated CD8+ T-cell activation by disturbing MHC I antigen binding, TCR signal transduction, and regulation of co-stimulators. In this way, noninflamed TME formed with inferior ICIs response and bad OS.

Apart from hyper-methylation of hub genes in T-cell activation, we also identified that hyper-methylation and downregulation of ARHGAP30 and ACAP1 as potential risk elements related to noninflamed TME. Although both methylation and expression level had no significant difference according to ICIs response, the important role of them in immunophenotype distinction could not be denied. The two elements both encode GAPs, which act by binding to the GTPase and promoting the conversion of small guanine nucleotide-binding proteins (small G proteins) from active GTP bound state to inactive GDP bound state. The representative substrate of GTPase are Ras family proteins, which are activated for participating in proliferation, attachment, motility, and promote malignancy. ARHGAP30 and ACAP1 are both GAPs for Ras superfamily proteins and past studies have demonstrated that over-expression of them attenuated malignant characteristics and acted as favorable prognostic factors [49, 50]. In our study, we obtained similar results and suggested that over-expression of the two genes were closely related to DNA hypo-methylation. As GAPs are downstream signal of T-cell activation, we supported the opinion that hypo-methylation of ARHGAP30 and ACAP1 were the outcomes of inflamed TME, instead of the reason.

In most of the ICIs clinical studies, patients that harbored oncogenic alternations were excluded [39]. Past studies showed that patients with sensitive mutations had lower TMB and immunogenicity compared with those wild-type patients [51]. Coinciding with the trend of the overall population, we found that the difference of the average methylation level was more pronounced according to immunophenotypes in the EGFR wt and KRAS wt subgroups. However, a smaller sample size of patients with oncogene mutation might also make sense. Methylation features of patients with or without TP53 alternations were similar. TP53 is a pro-oncogene, whose alternations are in the prophase of tumorigenesis and earlier than other epigenetic adaptation changes. In our study, we recognized EGFR/SRGN axis as a potential mechanism to discriminate ICIs response and OS. SRGN encodes a kind of proteoglycan, which can be secreted by the extracellular matrix of tumor cells to create a pro-inflammatory TME and is regarded as a driving factor of aggressive phenotype [52]. In our study, we illustrated that the over-expression of SRGN was closely regulated by hypo-methylation despite the EGFR status but the EGFR wt group tended to express more SRGN. EGFR wt patients accompanied with high expression of SRGN correlated with short OS but might benefit from ICIs. We demonstrated that EGFR wt with SRGN over-expression patients was displayed as inflamed tumors, but associated with “cancer-promoting inflammation”, which shaped TME toward a tumor-permissive state by chronic inflammation and immune evasion [53].

Our study had some limitations. Firstly, although we declared that mIMg played a crucial role in the immune response, and CD247, LCK, PSTPIP1, and SRGN were potential prognostic indicators, further analyses and large-scale clinical validations are needed. The association between mIMg and ICIs response deserves far more confirmations. Secondly, the measure of methylation beta value and expression data of every cohort might be influenced by many factors; in addition to samples' attributes and testing conditions, the threshold of methylation and expression level of different genes had distinct effects on results. In this condition, we set the cutoff value according to OS in every cohort, respectively, to reduce the batch effect. A consistent and steady cutoff value in the overall population may be needed. Besides, although prognostic ICIs response-related indicators were recognized, the direction of the association between immune response and methylation/expression status could not be determined. Past study demonstrated that ICIs could reshape the TCR repertoire, reiterating that TME was plastic and in continuous modification [54]. Hyper-methylation of CD247, LCK, and PSTPIP1 might be a way of TCR editing to influence immune response and survival. However, we could not determine whether it was hyper-methylation of TCR genes that led to immune exclusion or the immune surveillance that shaped or incapacitated TCR by DNA methylation. Moreover, oncogene mutations are numerous and in the dynamic change. We did not bring oncogenic alternations into multivariate Cox analysis in the process of choosing prognostic variables. To eliminate the effect of multicollinearity among the explanatory variables, we performed an extra hierarchical analysis according to oncogene status.

## 5. Conclusions

This study demonstrated the pivotal role of DNA methylation in immune response in LUAD. TCR editing by hyper-methylation of CD247, LCK, and PSTPIP1 acts as potential immune response indicators and prognostic factors. EGFR/SRGN axis involves in TME modification to influence clinical outcomes.

## Figures and Tables

**Table 1:** Multivariate Cox model of the prognostic molecular biomarkers by adjusting for age, gender, and disease stage.

Gene	TCGA			GPL570			GPL6884		
	HR^*∗*^	95%CI	*p*.val	HR^*∗*^	95%CI	*p*.val	HR^*∗*^	95%CI	*p*.val
ACAP1	1.49	1.07–2.07	0.019	1.43	0.96–2.12	0.076	2.02	1.38–2.95	<0.001
ARHGAP30	1.68	1.18–2.38	0.004	1.51	0.99–2.31	0.056	2.15	1.47–3.13	<0.001
CD247	1.68	1.14–2.48	0.009	1.44	1.01–2.03	0.042	2.17	1.49–3.17	<0.001
LAX1	1.49	1.01–2.2	0.045	2.02	1.37–2.98	<0.001	1.48	1.02–2.15	0.039
LCK	1.54	1.07–2.21	0.02	1.41	0.99–2.01	0.055	1.46	0.99–2.16	0.054
PSTPIP1	1.6	1.14–2.24	0.006	1.68	1.12–2.52	0.012	1.63	1.09–2.42	0.016

^*∗*^Low expression vs high expression.

## Data Availability

Publicly available datasets were analyzed in this study. The details and downloading websites of these data can be found in Table S1. The bioinformatic process is accessible on Github (https://github.com/HU-ZX/01_mIMg_LUAD_2022).

## Abbreviation:

LUAD: Lung adenocarcinoma
ICIs: Immune checkpoint inhibitors
TME: Tumor microenvironment
TCR: T-cell receptor
IMaS: Immune activity scores
IMpS: Immunophenotype score
MHC: Major histocompatibility complex
CYT: Cytolytic activity
EGFR: Epidermal growth factor receptor
TP53: Tumor protein p53
TCGA: The cancer genome atlas
GEO: Gene expression omnibus
GSEA: Gene set enrichment analysis
GO: Gene oncology
OS: Overall survival time
ROC: Receiver operating characteristic.

## Supplementary Materials

Supplementary Materials Table S1: Information of data was utilized in our study. Table S2: Clinical pathological features of the training cohorts. Table S3: Clinical pathological features of validation cohorts. Table S4: Methylation probes and corresponding regulating genes which both are highly correlated with IMpS. Table S5: Methylation probes/gene pairs which are closely correlated with each other. Table S6: Features of mIMg in immune response in the training cohorts. Table S7-1: Comparison of the methylation level of probes highly correlated with IMpS between ICIs responders and nonresponders. Table S7-2: Comparison of the expression level of corresponding genes in Table S7-1 between ICIs responders and nonresponders. Table S8: Multivariate Cox analysis of methylation probes in mIMg pairs which both distinguished OS in the TCGA-LUAD cohort. Table S9: Prognostic methylation probes according to oncogenes status. Figure S1: PCA analysis showed the distinct methylation status of genes in mIMg in three immunophenotypes. mIMg was defined as DNA methylation probe/gene pairs, genes in which were immune response–related hub genes and closely regulated by DNA methylation. Figure S2: Correlation between cg09032544/CD247, cg07786657/CD247, cg11683242/LCK, cg26227523/PSTPIP1 in mIMg and 7 immune features in GSE60644 & GSE56044, GSE66863 & GSE66836 cohorts. Figure S3: Evaluation of CD247, LCK, and PSTPIP1 in predicting ICIs response. (A–C) No significant difference was observed in the comparison of CD247, LCK, and PSTPIP1 expression levels between ICIs responders and nonresponders in GSE135222. Figure S4 Validation of CD247, LCK, and PSTPIP1in predicting the clinical outcome. (A–C), (G–I) Comparison of overall survival (OS) between groups based on the expression level of CD247, LCK, and PSTPIP1 in the GPL570 integrative cohort and GPL884 integrative cohort. Hazard ratio (HR) was calculated by univariate Cox analysis of methylation/expression level group. C-index was calculated based on univariate Cox model of methylation/expression level group. (D–F), (J–L) ROC curves of multivariate Cox models combining molecular stratification factors (CD247, LCK, PSTPIP1), age, gender, stage in the GPL570 integrative cohort and GPL884 integrative cohort. Figure S5: Decision curve analysis between the univariate Cox model of prognosis stratification factors including CD247, LCK, and PSTPIP1, and multivariate Cox model by adjusting for age, gender, stage in the TCGA-LUAD cohort and the GPL6884 integrative cohort. Figure S6: The potential role of ACAP1 and ARHGAP30 in predicting clinical outcome. (A) Correlation between cg252671438/ACAP1, cg01774645/ARHGAP30 in mIMg and 7 immune features in the TCGA-LUAD cohort. (B–E) Comparison of methylation/expression level of cg252671438/ACAP1, cg01774645/ARHGAP30 between ICIs responders and nonresponders in the GSE119144 & GSE135222 cohorts. (F)–(I) Comparison of overall survival (OS) between groups based on methylation/expression level of cg252671438/ACAP1, cg01774645/ARHGAP30 in the TCGA-LUAD cohort. (G–M) Comparison of overall survival (OS) between groups based on the expression level of ACAP1, ARHGAP30 in the GPL570 integrative cohort and the GPL884 integrative cohort. Hazard ratio (HR) in (F–M) was calculated by univariate Cox analysis of the methylation/expression level group. Figure S7: Crosstalk between methylation analysis and oncogene status. (A–F) Comparison of the average methylation level among different immune clusters stratified by oncogene status in the TCGA-LUAD cohort and the GSE66863 & GSE66836 cohorts. (G–I) Excavation of potential prognostic factors according to oncogene status in LUAD.
