# Supplementary material for: Hyper-Methylated Hub Genes of T-Cell Receptor Signaling Predict a Poor Clinical Outcome in Lung Adenocarcinoma
Source: J Oncol. 2022 Apr 6;2022:5426887. doi: 10.1155/2022/5426887 (PMC9007647; doi:10.1155/2022/5426887)
Supplement: Supplementary Materials — Table S1: Information of data was utilized in our study. Table S2: Clinical pathological features of the training cohorts. Table S3: Clinical pathological features of validation cohorts. Table S4: Methylation probes and corresponding regulating genes which both are highly correlated with IMpS. Table S5: Methylation probes/gene pairs which are closely correlated with each other. Table S6: Features of mIMg in immune response in the training cohorts. Table S7-1: Comparison of the methylation level of probes highly correlated with IMpS between ICIs responders and nonresponders. Table S7-2: Comparison of the expression level of corresponding genes in Table S7-1 between ICIs responders and nonresponders. Table S8: Multivariate Cox analysis of methylation probes in mIMg pairs which both distinguished OS in the TCGA-LUAD cohort. Table S9: Prognostic methylation probes according to oncogenes status. Figure S1: PCA analysis showed the distinct methylation status of genes in mIMg in three immunophenotypes. mIMg was defined as DNA methylation probe/gene pairs, genes in which were immune response–related hub genes and closely regulated by DNA methylation. Figure S2: Correlation between cg09032544/CD247, cg07786657/CD247, cg11683242/LCK, cg26227523/PSTPIP1 in mIMg and 7 immune features in GSE60644 & GSE56044, GSE66863 & GSE66836 cohorts. Figure S3: Evaluation of CD247, LCK, and PSTPIP1 in predicting ICIs response. (A–C) No significant difference was observed in the comparison of CD247, LCK, and PSTPIP1 expression levels between ICIs responders and nonresponders in GSE135222. Figure S4 Validation of CD247, LCK, and PSTPIP1in predicting the clinical outcome. (A–C), (G–I) Comparison of overall survival (OS) between groups based on the expression level of CD247, LCK, and PSTPIP1 in the GPL570 integrative cohort and GPL884 integrative cohort. Hazard ratio (HR) was calculated by univariate Cox analysis of methylation/expression level group. C-index was calculated based on univariate Co [file 5426887.f1.zip › 5426887.f1/Figure_S3.pdf]

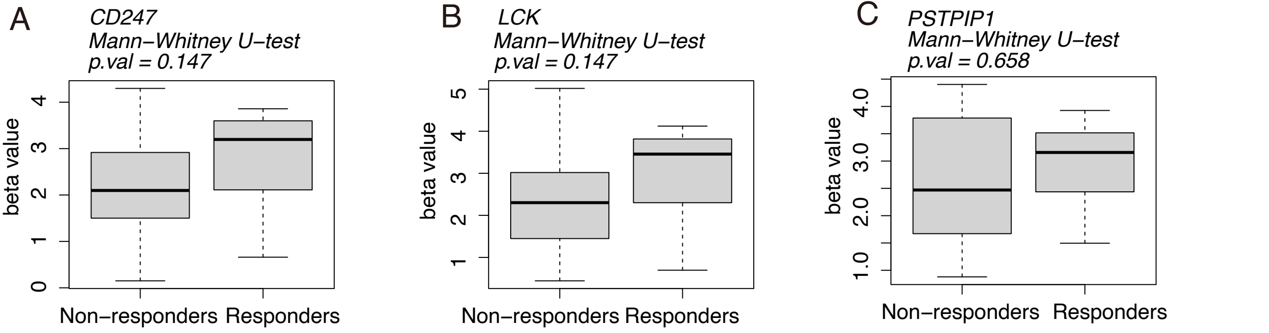

A
CD247
Mann−Whitney U−test
p.val = 0.147
beta value
0
1
2
3
4
Non−responders
Responders
B
LCK
Mann−Whitney U−test
p.val = 0.147
beta value
1
2
3
4
5
Non−responders
Responders
C
PSTPIP1
Mann−Whitney U−test
p.val = 0.658
beta value
1.0
2.0
3.0
4.0
Non−responders
Responders

Figure S3
