# Supplementary material for: Hyper-Methylated Hub Genes of T-Cell Receptor Signaling Predict a Poor Clinical Outcome in Lung Adenocarcinoma
Source: J Oncol. 2022 Apr 6;2022:5426887. doi: 10.1155/2022/5426887 (PMC9007647; doi:10.1155/2022/5426887)
Supplement: Supplementary Materials — Table S1: Information of data was utilized in our study. Table S2: Clinical pathological features of the training cohorts. Table S3: Clinical pathological features of validation cohorts. Table S4: Methylation probes and corresponding regulating genes which both are highly correlated with IMpS. Table S5: Methylation probes/gene pairs which are closely correlated with each other. Table S6: Features of mIMg in immune response in the training cohorts. Table S7-1: Comparison of the methylation level of probes highly correlated with IMpS between ICIs responders and nonresponders. Table S7-2: Comparison of the expression level of corresponding genes in Table S7-1 between ICIs responders and nonresponders. Table S8: Multivariate Cox analysis of methylation probes in mIMg pairs which both distinguished OS in the TCGA-LUAD cohort. Table S9: Prognostic methylation probes according to oncogenes status. Figure S1: PCA analysis showed the distinct methylation status of genes in mIMg in three immunophenotypes. mIMg was defined as DNA methylation probe/gene pairs, genes in which were immune response–related hub genes and closely regulated by DNA methylation. Figure S2: Correlation between cg09032544/CD247, cg07786657/CD247, cg11683242/LCK, cg26227523/PSTPIP1 in mIMg and 7 immune features in GSE60644 & GSE56044, GSE66863 & GSE66836 cohorts. Figure S3: Evaluation of CD247, LCK, and PSTPIP1 in predicting ICIs response. (A–C) No significant difference was observed in the comparison of CD247, LCK, and PSTPIP1 expression levels between ICIs responders and nonresponders in GSE135222. Figure S4 Validation of CD247, LCK, and PSTPIP1in predicting the clinical outcome. (A–C), (G–I) Comparison of overall survival (OS) between groups based on the expression level of CD247, LCK, and PSTPIP1 in the GPL570 integrative cohort and GPL884 integrative cohort. Hazard ratio (HR) was calculated by univariate Cox analysis of methylation/expression level group. C-index was calculated based on univariate Co [file 5426887.f1.zip › 5426887.f1/Table S1-3.pdf]

Table S1 Information of data utilized in our study

Cohort	Downloading website	Function	Samples	Platform	Expression	Methylation	Mutation	ICIs response	Overall survival
TCGA-LUAD	https://portal.gdc.cancer.gov/projects/TCGA-LUAD	Training	450	Illumina HiSeq/ Illumina HumanMethylation450 BeadChip	√	√	√	×	√
GSE60644	https://www.ncbi.nlm.nih.gov/geo/query/acc.cgi?acc =GSE60644	Training	78	Illumina HumanHT-12 V4.0 expression beadchip	√	×	×	×	×
GSE56044	https://www.ncbi.nlm.nih.gov/geo/query/acc.cgi?acc =GSE56044	Training	78	Illumina HumanMethylation450 BeadChip	×	√	×	×	×
GSE66863	https://www.ncbi.nlm.nih.gov/geo/query/acc.cgi?acc =GSE66863	Training	112	Agilent-028004 SurePrint G3 Human GE 8x60K Microarray	√	×	√	×	×
GSE66836	https://www.ncbi.nlm.nih.gov/geo/query/acc.cgi?acc =GSE66836	Training	112	Illumina HumanMethylation450 BeadChip	×	√	√	×	×
GSE37745	https://www.ncbi.nlm.nih.gov/geo/query/acc.cgi?acc =GSE37745	Validation	92	[HG-U133_Plus_2] Affymetrix Human Genome U133 Plus 2.0 Array	√	×	×	×	√
GSE50081	https://www.ncbi.nlm.nih.gov/geo/query/acc.cgi?acc =GSE50081	Validation	112	[HG-U133_Plus_2] Affymetrix Human Genome U133 Plus 2.0 Array	√	×	×	×	√

GSE14814	https://www.ncbi.nlm.nih.gov/geo/query/acc.cgi?acc=GSE14814	Validation	68	[HG-U133A] Affymetrix Human Genome U133A Array	√	×	×	×	√
GSE41271	https://www.ncbi.nlm.nih.gov/geo/query/acc.cgi?acc=GSE41271	Validation	183	Illumina HumanWG-6 v3.0 expression beadchip	√	×	×	×	√
GSE42127	https://www.ncbi.nlm.nih.gov/geo/query/acc.cgi?acc=GSE42127	Validation	133	Illumina HumanWG-6 v3.0 expression beadchip	√	×	×	×	√
GSE13213	https://www.ncbi.nlm.nih.gov/geo/query/acc.cgi?acc=GSE13213	Validation	117	Agilent-014850 Whole Human Genome Microarray 4x44K G4112F	√	×	√	×	√
GSE11969	https://www.ncbi.nlm.nih.gov/geo/query/acc.cgi?acc=GSE11969	Validation	90	Agilent Homo sapiens 21.6K custom array	√	×	√	×	√
GSE135222	https://www.ncbi.nlm.nih.gov/geo/query/acc.cgi?acc=GSE135222	Validation	27	Illumina HiSeq	√	×	×	√	×
GSE119144	https://www.ncbi.nlm.nih.gov/geo/query/acc.cgi?acc=GSE119144	Validation	58	Illumina Infinium HumanMethylation850 BeadChip	×	√	×	√	×

Table S2-1 Clinical pathological features of TCGA-LUAD cohort

	[ALL] N=450	Cluster1 N=247	Cluster2 N=166	Cluster3 N=37	p.overall
Age:					0.083
<65	204 (47.4%)	115 (48.5%)	66 (42.3%)	23 (62.2%)	
>=65	226 (52.6%)	122 (51.5%)	90 (57.7%)	14 (37.8%)	
Gender:					0.47
female	242 (53.8%)	131 (53.0%)	94 (56.6%)	17 (45.9%)	
male	208 (46.2%)	116 (47.0%)	72 (43.4%)	20 (54.1%)	
Stage:					.
Stage I	244 (54.7%)	125 (51.0%)	98 (59.8%)	21 (56.8%)	
Stage II	109 (24.4%)	62 (25.3%)	39 (23.8%)	8 (21.6%)	
Stage III	73 (16.4%)	49 (20.0%)	21 (12.8%)	3 (8.11%)	
Stage IV	20 (4.48%)	9 (3.67%)	6 (3.66%)	5 (13.5%)	
T_Stage:					.
T1	152 (33.8%)	86 (34.8%)	60 (36.1%)	6 (16.2%)	
T2	240 (53.3%)	126 (51.0%)	89 (53.6%)	25 (67.6%)	
T3	39 (8.67%)	25 (10.1%)	11 (6.63%)	3 (8.11%)	
T4	16 (3.56%)	8 (3.24%)	5 (3.01%)	3 (8.11%)	
TX	3 (0.67%)	2 (0.81%)	1 (0.60%)	0 (0.00%)	
N_Stage:					.
N0	293 (65.1%)	151 (61.1%)	113 (68.1%)	29 (78.4%)	
N1	81 (18.0%)	46 (18.6%)	30 (18.1%)	5 (13.5%)	
N2	65 (14.4%)	43 (17.4%)	19 (11.4%)	3 (8.11%)	
N3	1 (0.22%)	1 (0.40%)	0 (0.00%)	0 (0.00%)	
NX	10 (2.22%)	6 (2.43%)	4 (2.41%)	0 (0.00%)	
M_Stage:					0.081
M0	289 (64.2%)	165 (66.8%)	104 (62.7%)	20 (54.1%)	
M1	19 (4.22%)	9 (3.64%)	5 (3.01%)	5 (13.5%)	
MX	142 (31.6%)	73 (29.6%)	57 (34.3%)	12 (32.4%)	
Smoking history:					0.249
< 30py	109 (35.4%)	55 (32.0%)	46 (41.4%)	8 (32.0%)	
>= 30py	199 (64.6%)	117 (68.0%)	65 (58.6%)	17 (68.0%)	
EGFR status:					0.208
mutated	59 (13.1%)	33 (13.4%)	18 (10.8%)	8 (21.6%)	
wild type	391 (86.9%)	214 (86.6%)	148 (89.2%)	29 (78.4%)	
KRAS status:					0.01
mutated	111 (24.7%)	74 (30.0%)	28 (16.9%)	9 (24.3%)	
wild type	339 (75.3%)	173 (70.0%)	138 (83.1%)	28 (75.7%)	

Tp53 status:					0.922
mutated	217 (48.2%)	121 (49.0%)	78 (47.0%)	18 (48.6%)	
wild type	233 (51.8%)	126 (51.0%)	88 (53.0%)	19 (51.4%)	

Table S2-2 Clinical pathological features of GSE66863 & GSE66836 cohort

	[ALL] N=112	Cluster1 N=69	Cluster2 N=23	Cluster3 N=20	p.overall
Age:					0.328
<65	51 (45.9%)	35 (51.5%)	9 (39.1%)	7 (35.0%)	
>=65	60 (54.1%)	33 (48.5%)	14 (60.9%)	13 (65.0%)	
Gender:					0.728
Female	59 (52.7%)	37 (53.6%)	13 (56.5%)	9 (45.0%)	
Male	53 (47.3%)	32 (46.4%)	10 (43.5%)	11 (55.0%)	
Stage:					0.372
I	66 (58.9%)	35 (50.7%)	18 (78.3%)	13 (65.0%)	
II	23 (20.5%)	16 (23.2%)	3 (13.0%)	4 (20.0%)	
III	22 (19.6%)	17 (24.6%)	2 (8.70%)	3 (15.0%)	
IV	1 (0.89%)	1 (1.45%)	0 (0.00%)	0 (0.00%)	
Smoking history:					0.85
Never	14 (12.5%)	9 (13.0%)	2 (8.70%)	3 (15.0%)	
Smoker	98 (87.5%)	60 (87.0%)	21 (91.3%)	17 (85.0%)	
EGFR status:					0.39
mutated	15 (13.6%)	11 (16.4%)	1 (4.35%)	3 (15.0%)	
wild type	95 (86.4%)	56 (83.6%)	22 (95.7%)	17 (85.0%)	
KRAS status:					0.85
mutated	36 (33.6%)	23 (33.3%)	8 (38.1%)	5 (29.4%)	
wild type	71 (66.4%)	46 (66.7%)	13 (61.9%)	12 (70.6%)	
Tp53 status:					0.816
mutated	45 (41.3%)	28 (42.4%)	10 (43.5%)	7 (35.0%)	
wild type	64 (58.7%)	38 (57.6%)	13 (56.5%)	13 (65.0%)	

Table S2-3 Clinical pathological features of GSE60644 & GSE56044 cohort

	[ALL] N=78	Cluster1 N=36	Cluster2 N=31	Cluster3 N=11	p.overall
Age:					0.097
<65	31 (40.3%)	10 (28.6%)	14 (45.2%)	7 (63.6%)	
>=65	46 (59.7%)	25 (71.4%)	17 (54.8%)	4 (36.4%)	
Gender:					0.207
Female	45 (57.7%)	24 (66.7%)	17 (54.8%)	4 (36.4%)	

Male	33 (42.3%)	12 (33.3%)	14 (45.2%)	7 (63.6%)

Table S3-1 Clinical pathological features of GPL570 combined cohort

	[ALL] N=272	N
Age:		272
< 65	131 (48.2%)	
>= 65	141 (51.8%)	
Gender:		272
Female	138 (50.7%)	
Male	134 (49.3%)	
Stage:		272
StageI-II	260 (95.6%)	
StageIII-IV	12 (4.41%)	
T stage:		112
T1	37 (33.0%)	
T2	73 (65.2%)	
T3	2 (1.79%)	
N stage:		112
N0	85 (75.9%)	
N1	27 (24.1%)	
M stage:		112
M0	112 (100%)	
Smoking history:		112
Current	33 (29.5%)	
Ex-smoker	49 (43.8%)	
Never	20 (17.9%)	
Unabletodetermine	10 (8.93%)	
GSE:		272
GSE14814	68 (25.0%)	
GSE37745	92 (33.8%)	
GSE50081	112 (41.2%)	

Table S3-2 Clinical pathological features of GPL6884 combined cohort

	[ALL] N=316	N
Age:		315
< 65	158 (50.2%)	
>= 65	157 (49.8%)	
Gender:		316

Female	155 (49.1%)	
Male	161 (50.9%)	
Stage:		316
StageI-II	240 (75.9%)	
StageIII-IV	76 (24.1%)	
Smoking history:		182
Yes	26 (14.3%)	
No	156 (85.7%)	
GSE:		316
GSE41271	183 (57.9%)	
GSE42127	133 (42.1%)	

Table S3-3 Clinical pathological features of GSE13213

	[ALL] N=117	N
Age:		117
<65	76 (65.0%)	
>=65	41 (35.0%)	
Gender:		117
Female	57 (48.7%)	
Male	60 (51.3%)	
Smoking history		117
No	56 (47.9%)	
Yes	61 (52.1%)	
T stage:		117
T1	54 (46.2%)	
T2	50 (42.7%)	
T3	8 (6.84%)	
T4	5 (4.27%)	
N stage:		117
N0	87 (74.4%)	
N1	8 (6.84%)	
N2	22 (18.8%)	
M stage:		117
M0	117 (100%)	
Stage:		117
StageI-II	92 (78.6%)	
StageIII-IV	25 (21.4%)	
EGFR status:		117
mutated	45 (38.5%)	

wild type	72 (61.5%)	
KRAS status:		117
mutated	15 (12.8%)	
wild type	102 (87.2%)	
Tp53 status:		116
mutated	38 (32.8%)	
wild type	78 (67.2%)	

Table S3-4 Clinical pathological features of GSE11969

	[ALL] N=90	N
Age:		90
<65	58 (64.4%)	
>=65	32 (35.6%)	
Gender:		90
Female	43 (47.8%)	
Male	47 (52.2%)	
Smoking:		90
No	45 (50.0%)	
Yes	45 (50.0%)	
T stage:		90
T1	40 (44.4%)	
T2	37 (41.1%)	
T3	8 (8.89%)	
T4	5 (5.56%)	
N stage:		90
N0	60 (66.7%)	
N1	8 (8.89%)	
N2	22 (24.4%)	
M stage:		90
M0	90 (100%)	
Stage:		90
StageI-II	65 (72.2%)	
StageIII-IV	25 (27.8%)	
EGFR status:		90
mutated	32 (35.6%)	
wild type	58 (64.4%)	
KRAS status:		90
mutated	10 (11.1%)	
wild type	80 (88.9%)	

Tp53 status:		90
mutated	29 (32.2%)	
wild type	61 (67.8%)	
