# Supplementary material for: Hyper-Methylated Hub Genes of T-Cell Receptor Signaling Predict a Poor Clinical Outcome in Lung Adenocarcinoma
Source: J Oncol. 2022 Apr 6;2022:5426887. doi: 10.1155/2022/5426887 (PMC9007647; doi:10.1155/2022/5426887)
Supplement: Supplementary Materials — Table S1: Information of data was utilized in our study. Table S2: Clinical pathological features of the training cohorts. Table S3: Clinical pathological features of validation cohorts. Table S4: Methylation probes and corresponding regulating genes which both are highly correlated with IMpS. Table S5: Methylation probes/gene pairs which are closely correlated with each other. Table S6: Features of mIMg in immune response in the training cohorts. Table S7-1: Comparison of the methylation level of probes highly correlated with IMpS between ICIs responders and nonresponders. Table S7-2: Comparison of the expression level of corresponding genes in Table S7-1 between ICIs responders and nonresponders. Table S8: Multivariate Cox analysis of methylation probes in mIMg pairs which both distinguished OS in the TCGA-LUAD cohort. Table S9: Prognostic methylation probes according to oncogenes status. Figure S1: PCA analysis showed the distinct methylation status of genes in mIMg in three immunophenotypes. mIMg was defined as DNA methylation probe/gene pairs, genes in which were immune response–related hub genes and closely regulated by DNA methylation. Figure S2: Correlation between cg09032544/CD247, cg07786657/CD247, cg11683242/LCK, cg26227523/PSTPIP1 in mIMg and 7 immune features in GSE60644 & GSE56044, GSE66863 & GSE66836 cohorts. Figure S3: Evaluation of CD247, LCK, and PSTPIP1 in predicting ICIs response. (A–C) No significant difference was observed in the comparison of CD247, LCK, and PSTPIP1 expression levels between ICIs responders and nonresponders in GSE135222. Figure S4 Validation of CD247, LCK, and PSTPIP1in predicting the clinical outcome. (A–C), (G–I) Comparison of overall survival (OS) between groups based on the expression level of CD247, LCK, and PSTPIP1 in the GPL570 integrative cohort and GPL884 integrative cohort. Hazard ratio (HR) was calculated by univariate Cox analysis of methylation/expression level group. C-index was calculated based on univariate Co [file 5426887.f1.zip › 5426887.f1/Table S9.pdf]

TableS9-1 Prognostic methylation probes in EGFR wt group of TCGA-LUAD cohort

Probe	HR *	95%CI	P.val	gene
cg09721047	0.66	0.44-0.99	0.046	ARHGAP25
cg01774645	0.61	0.41-0.91	0.015	ARHGAP30
cg20009327	0.54	0.37-0.78	0.001	ARHGAP9
cg05584759	0.66	0.45-0.98	0.039	ARHGAP9
cg08799922	2.17	1.31-3.6	0.003	C1R
cg17067993	1.73	1.1-2.72	0.018	CCR7
cg07786657	0.62	0.42-0.93	0.021	CD247
cg02189760	0.65	0.45-0.94	0.021	CD37
cg20644754	0.61	0.41-0.9	0.012	CD37
cg07542476	0.66	0.45-0.95	0.026	CD37
cg07575373	0.62	0.43-0.89	0.01	CD37
cg15046675	0.64	0.43-0.95	0.025	CD37
cg07371747	1.61	1.06-2.43	0.024	CIITA
cg16277944	0.51	0.32-0.82	0.006	DOCK2
cg13863007	1.68	1.06-2.68	0.028	FGD3
cg19014335	1.95	1.22-3.11	0.005	FMNL3
cg19109120	0.43	0.29-0.63	0	HVCN1
cg16749930	0.6	0.41-0.88	0.009	IL18BP

cg09383860	0.52	0.34-0.8	0.003	IL18BP
cg16478536	0.58	0.39-0.86	0.007	IRF4
cg20425130	0.63	0.42-0.94	0.024	KLHL6
cg01765930	0.66	0.45-0.97	0.036	MFNG
cg18126557	0.62	0.42-0.91	0.016	NLRP3
cg01943221	0.6	0.39-0.91	0.015	PIK3CD
cg09031790	0.64	0.41-0.98	0.041	PRKCB
cg18505752	0.56	0.36-0.87	0.01	PSMB8
cg25693349	0.63	0.43-0.92	0.017	PSMB8
cg02841844	1.73	1.05-2.87	0.033	RASSF4
cg20781880	0.67	0.45-0.99	0.046	S1PR4
cg02851793	2.25	1.46-3.48	0	SRGN
cg01903374	0.52	0.31-0.87	0.012	TNFAIP8L2
cg21773162	0.64	0.43-0.95	0.027	ZAP70

* Hypo-methylation vs hyper-methylation

TableS9-2 Prognostic methylation probes in EGFR mut group of TCGA-LUAD cohort

Probe	HR *	95%CI	P.val	gene
cg25671438	0.36	0.13-0.98	0.045	ACAP1
cg25039325	0.27	0.1-0.75	0.012	ARHGAP25
cg26238936	0.21	0.07-0.7	0.011	ARHGAP25
cg01774645	0.19	0.04-0.86	0.03	ARHGAP30
cg15880738	0.35	0.14-0.89	0.027	CD3G
cg19109120	3.24	1.21-8.67	0.019	HVCN1
cg01493517	0.21	0.05-0.82	0.024	IFFO1
cg20425130	0.3	0.1-0.9	0.032	KLHL6
cg15254671	0.22	0.05-0.99	0.049	MYO1F
cg01943221	0.2	0.05-0.81	0.024	PIK3CD
cg15293582	0.32	0.12-0.85	0.023	PRF1
cg02841844	0.12	0.01-0.98	0.048	RASSF4
cg25765104	0.37	0.15-0.95	0.038	RCSD1
cg05242653	0.21	0.07-0.64	0.006	STX11
cg13165140	0.38	0.15-0.95	0.039	TAP1
cg12253437	0.36	0.14-0.91	0.031	TAP1
cg09354037	0.33	0.13-0.84	0.02	TAP1
cg05693489	0.35	0.13-0.89	0.028	TAP1

cg00336376	3.12	1.03-9.46	0.044	ZNF831

* Hypo-methylation vs hyper-methylation

TableS9-3 Prognostic methylation probes in KRAS wt group of TCGA-LUAD cohort

Probe	HR *	95%CI	P.val	gene
cg19175364	1.9	1.2-3	0.006	ACAP1
cg25900902	1.68	1.02-2.75	0.04	ACAP1
cg25671438	0.49	0.32-0.76	0.001	ACAP1
cg25039325	0.43	0.27-0.67	0	ARHGAP25
cg09721047	0.53	0.34-0.82	0.004	ARHGAP25
cg26238936	0.52	0.34-0.81	0.004	ARHGAP25
cg01774645	0.45	0.29-0.7	0	ARHGAP30
cg08799922	2.38	1.4-4.04	0.001	C1R
cg17067993	0.47	0.28-0.8	0.005	CCR7
cg09032544	0.56	0.37-0.83	0.005	CD247
cg07786657	0.51	0.34-0.76	0.001	CD247
cg03724640	0.54	0.34-0.87	0.011	CD27
cg11384427	0.46	0.3-0.7	0	CD27
cg15046675	0.53	0.35-0.82	0.004	CD37
cg07728874	0.55	0.35-0.87	0.01	CD3D

cg04602992	0.52	0.33-0.82	0.005	CD79B
cg07371747	1.59	1.02-2.46	0.041	CIITA
cg19109120	0.5	0.33-0.76	0.001	HVCN1
cg01493517	0.44	0.27-0.71	0.001	IFFO1
cg01979888	0.49	0.29-0.81	0.005	IFFO1
cg16749930	0.38	0.24-0.59	0	IL18BP
cg09383860	0.41	0.26-0.64	0	IL18BP
cg20425130	0.53	0.34-0.83	0.005	KLHL6
cg09606470	0.52	0.3-0.91	0.022	KLHL6
cg17169196	0.54	0.34-0.86	0.009	LTA
cg08230957	0.57	0.34-0.96	0.033	MAP4K1
cg22987448	0.4	0.2-0.79	0.008	MYO1F
cg08283130	1.85	1.07-3.19	0.027	MYO1F
cg04865264	3.07	1.67-5.64	0	OVOL2
cg23262134	1.95	1.06-3.57	0.031	OVOL2
cg17832991	1.94	1.26-2.99	0.002	OVOL2
cg11381564	0.5	0.32-0.76	0.001	PSMB8
cg19136673	0.57	0.36-0.89	0.014	PSMB8
cg18505752	0.49	0.32-0.75	0.001	PSMB8
cg25693349	0.46	0.29-0.72	0.001	PSMB8
cg23095383	0.56	0.38-0.84	0.005	PTPRC

cg01062942	1.66	1.03-2.7	0.039	RASAL3
cg11264392	1.78	1.08-2.93	0.024	RASAL3
cg27125093	0.6	0.38-0.94	0.026	S1PR4
cg20781880	0.56	0.36-0.86	0.009	S1PR4
cg15518883	0.41	0.25-0.67	0	SIT1
cg13876315	0.57	0.38-0.86	0.008	SIT1
cg03699843	0.57	0.36-0.89	0.014	SNX20
cg08330349	0.52	0.33-0.82	0.005	SNX20
cg01144086	0.61	0.39-0.94	0.026	SNX20
cg14614581	0.48	0.32-0.73	0.001	SPOCK2
cg26033526	0.66	0.44-0.99	0.046	TAP1
cg09354037	0.61	0.39-0.97	0.035	TAP1
cg05693489	0.64	0.41-0.98	0.042	TAP1
cg10402417	0.33	0.18-0.59	0	TBC1D10C
cg15698795	0.52	0.33-0.81	0.004	TBC1D10C
cg11600161	0.43	0.26-0.73	0.002	TBC1D10C
cg00862408	0.56	0.36-0.86	0.009	TBC1D10C
cg01903374	0.54	0.35-0.84	0.007	TNFAIP8L2
cg00336376	1.83	1.22-2.75	0.003	ZNF831

* Hypo-methylation vs hyper-methylation

TableS9-4 Prognostic methylation probes in KRAS mut group of TCGA-LUAD cohort

Probe	HR	95%CI	P.val	gene
cg20009327	0.42	0.21-0.85	0.015	ARHGAP9
cg17067993	8.58	1.15-63.85	0.036	CCR7
cg20644754	0.37	0.17-0.83	0.016	CD37
cg15046675	0.41	0.19-0.89	0.023	CD37
cg10981907	2.36	1.05-5.33	0.039	CD6
cg19109120	0.15	0.04-0.59	0.007	HVCN1
cg24859236	0.22	0.07-0.72	0.013	PIK3CD
cg23251761	0.18	0.06-0.6	0.005	PIK3CD
cg26227523	3.31	1.13-9.68	0.029	PSTPIP1
cg00336376	0.1	0.01-0.76	0.026	ZNF831

* Hypo-methylation vs hyper-methylation

TableS9-5 Prognostic methylation probes in Tp53 wt group of TCGA-LUAD cohort

Probe	HR *	95%CI	P.val	gene
cg25671438	0.5	0.28-0.91	0.023	ACAP1
cg05584759	0.35	0.16-0.76	0.007	ARHGAP9
cg07786657	0.51	0.29-0.91	0.023	CD247
cg11384427	0.52	0.3-0.91	0.022	CD27
cg15880738	0.38	0.19-0.75	0.005	CD3G
cg20425130	0.47	0.25-0.88	0.018	KLHL6
cg06958535	0.47	0.26-0.82	0.008	LAX1
cg20654468	0.47	0.23-0.97	0.041	LPXN
cg09031790	0.45	0.25-0.84	0.012	PRKCB
cg11381564	0.5	0.27-0.92	0.027	PSMB8
cg19136673	0.41	0.22-0.76	0.005	PSMB8
cg18505752	0.52	0.3-0.9	0.02	PSMB8
cg25693349	0.48	0.26-0.89	0.019	PSMB8
cg15518883	0.49	0.28-0.86	0.013	SIT1
cg15844596	0.47	0.25-0.89	0.021	SLA2
cg07598052	0.44	0.22-0.86	0.016	SLA2
cg06340367	0.48	0.25-0.92	0.026	SLA2
cg02851793	2.88	1.5-5.54	0.002	SRGN

cg12253437	0.38	0.2-0.71	0.002	TAP1
cg09354037	0.5	0.25-0.97	0.041	TAP1
cg25384897	0.46	0.25-0.87	0.018	TAP1
cg05693489	0.43	0.24-0.79	0.006	TAP1
cg00336376	1.84	1.06-3.19	0.029	ZNF831

TableS9-6 Prognostic methylation probes in Tp53 mut group of TCGA-LUAD cohort

Probe	HR *	95%CI	P.val	gene
cg25039325	0.51	0.31-0.84	0.008	ARHGAP25
cg09721047	0.58	0.34-0.99	0.044	ARHGAP25
cg26238936	0.48	0.25-0.92	0.027	ARHGAP25
cg20009327	0.5	0.32-0.81	0.004	ARHGAP9
cg05584759	0.62	0.39-1	0.049	ARHGAP9
cg20644754	0.48	0.28-0.8	0.006	CD37
cg15880738	0.51	0.3-0.87	0.014	CD3G
cg07371747	1.91	1.02-3.56	0.042	CIITA
cg08453449	0.57	0.35-0.91	0.019	CMKLR1
cg16277944	0.53	0.32-0.89	0.016	DOCK2
cg23477774	0.49	0.31-0.79	0.004	DOCK2
cg00071250	0.39	0.23-0.67	0.001	FASLG
cg02556655	2.52	1.24-5.11	0.011	FERMT3

cg13863007	2.39	1.21-4.73	0.012	FGD3
cg19014335	2.2	1.21-3.99	0.01	FMNL3
cg19109120	0.44	0.27-0.71	0.001	HVCN1
cg15375424	2.35	1.27-4.34	0.007	IRF1
cg08230957	0.44	0.22-0.88	0.02	MAP4K1
cg15254671	2.04	1-4.16	0.049	MYO1F
cg18126557	0.53	0.31-0.89	0.017	NLRP3
cg01943221	0.42	0.21-0.83	0.013	PIK3CD
cg15027815	2.35	1.17-4.71	0.016	PTPN7
cg01062942	0.48	0.27-0.85	0.011	RASAL3
cg14614581	0.51	0.29-0.89	0.018	SPOCK2
cg00862408	2.1	1.15-3.83	0.015	TBC1D10C
cg11791526	2.02	1.25-3.27	0.004	TBC1D10C
cg12050508	0.56	0.33-0.94	0.03	TNFAIP3

* Hypo-methylation vs hyper-methylation
